# *BpWOX11* promotes adventitious root formation in* Betula pendula*

**DOI:** 10.1186/s12870-023-04703-z

**Published:** 2024-01-02

**Authors:** Kun Chen, Xiaoyue Zhang, Zhenglun Li, Wei Wang, Guanbin Lv, Qibin Yu, Guifeng Liu, Chuanping Yang, Jing Jiang

**Affiliations:** 1 State Key Laboratory of Tree Genetics And Breeding, Northeast Forestry University, 26 Hexing Road, Harbin, 150040 China; 2 Citrus Research and Education Center, University of Florida, Lake Alfred, FL 33850 USA

**Keywords:** *Betula pendula*, *BpWOX11*, Cuttings, Adventitious roots, Cell division, Stress response

## Abstract

Adventitious root formation is a key step in vegetative propagation via cuttings. It is crucial for establishing birch plantations and preserve birch varieties. Although previous studies have highlighted role of *WOX11* in controlling adventitious root formation, no such study has been conducted in birch. Understanding the mechanism of adventitious root formation is essential for improvement of rooting or survival rate using stem cuttings in birch. In this study, we cloned *BpWOX11* and produced *BpWOX11* overexpression (OE) transgenic lines using the *Agrobacterium*-mediated plant transformation. OE lines exhibited early initiated adventitious root formation, leading to increase the rooting rate of stem cuttings plants. RNA sequencing analysis revealed that OE lines induced the gene expression related to expansin and cell division pathway, as well as defense and stress response genes. These may be important factors for the *BpWOX11* gene to promote adventitious root formation in birch cuttings. The results of this study will help to further understand the molecular mechanisms controlling the formation of adventitious roots in birch.

**Supplementary Information:**

The online version contains supplementary material available at 10.1186/s12870-023-04703-z.

## Introduction

Vegetative propagation using cuttings is an important method for forest reproduction. The induction of explants to produce adventitious roots (ARs) is a key step for developing full plant [1–3]. Enhancing success rate relies on promoting the formation and development of ARs in vitro. In vitro plant propagation, external conditions such as temperature, humidity, and hormone levels play crucial roles in influencing AR formation and development [4–8]. In addition to external factors, differences in gene expression can affect the process of isolated rooting (from head organogenesis) in plants [9]. Notably, *LkARF7* and *LkARF19* have been identified as promoting AR formation in heterologous poplar by positively regulating *LkBBM1* [10]. Overexpressing *AP2* family transcription factor AINTEGUMENTA LIKE1 increased the number of ARs in *Populus trichocarpa* [11]. Additionally, WUSCHEL (WUS)-related homeobox (WOX) genes play an essential role in coordinating the transcription of genes involved in the function and organogenesis of stem and root meristems [12].

WOX is a family of specific plant transcription factors crucial in developmental processes in plants such as embryonic pattern, stem cell maintenance, and organ formation [13]. WOX proteins can be divided in three major clades according to phylogenetic analysis: the WUS, intermediate, and ancient branches [13]. The intermediate branch includes WOX8, WOX9, WOX11, and WOX12 proteins, and *WOX11* plays an essential role in AR formation in plants. A loss-of-function mutation in *WOX11* leads to a decrease in the number of crown roots (a type of AR) and growth rate in rice (*Oryza sativa*). The overexpression (OE) of this gene promotes the early occurrence of crown roots and significantly increases root biomass [14]. In addition, the presence of the transcription factor WOX11 was required for crown root proliferation induced in *OsYUCs*(*OsYUC3*, *OsYUC4*, *OsYUC8*, *OsYUC10*, *OsYUC11* and *OsYUC14*) overexpression [15, 16]. Inhibition of the *WOX11* leads to decrease rooting ability, whereas *WOX11* OE increases root formation in *Arabidopsis* [2, 9]. *WOX11* directly affects damage-induced auxin accumulation in the cambium and its surroundings, upregulates the expression of *LBD16* and *LBD29*, and transforms the leaf cambium or its nearby parenchyma cells into root cells [9]. The OE of *PeWOX11a* or *PeWOX11b* not only increases the number of ARs on the cuttings but also induces the formation of ectopic roots in the aboveground parts in *Populus* × *euramericana* [17]. In contrast, *LncWOX11a* may inhibit the development of ARs by downregulating *PeWOX11a* [18]. Therefore, *de novo* organ regeneration studies have identified *WOX11* as a key gene involved in cell fate transition. *WOX11* increases the number of ARs and promotes their formation in isolated plant organs [9, 19].

Due to its low cuttings rooting rate, *Betula pendula* faces challenges in expanding vegetative propagation through cuttings. Although *WOX11* has been extensively studied in *Arabidopsis*, rice, and *Populus trichocarpa*, functional study of *WOX11* in birch is scarce. In this study, we identified the cDNA sequence of *BpWOX11* and successfully obtained *WOX11* OE transgenic lines. We investigated the rooting index of transgenic lines and performed RNA sequencing (RNA-seq) to identify the differentially expressed genes (DEGs) related to ARs. Our study provides insight into molecular mechanism of ARs and offers the potential application to increase the rooting rate of birch plants through cuttings.

## Materials and methods

### Plant material

These birch (*Betula pendula)* plants were planted in the Seed Research of Northeast Forestry University (126° 64′ E, 45° 72′ N) in Northeast Forestry University, Harbin, China. The mature fruit was dried; the bracts and pedicels were removed. The seeds were sealed and stored in a refrigerator at − 20 °C. *BpWOX11* was cloned from birch *and* transferred to mature birch seeds.

### Cloning and bioinformatics analysis of *BpWOX11*

The sequence of *AtWOX11* was retrieved from the *Arabidopsis thaliana* database (https://www.arabidopsis.org), and homologous sequence was identified in the birch genome. Based on the mRNA sequence, the upstream and downstream primers of *BpWOX11* were designed (Table S1). The cDNA of the wild-type (WT) birch cuttings was used as a template for PCR amplification. The PCR product was purified and ligated using the pCloneEZ vector (pCloneEZ-*BpWOX11*). After transformation, the monoclonal clones were selected for sequencing at Beijing Qingke Biotechnology Co. Ltd.

Protein coding sequence (CDS) of *BpWOX11* was obtained from reference genome of *Betula pendula*. Gene sequences are available at COGE (https://genomevolution.org/coge/) using the login number: *BpWOX11* (Bpev01.c0441.g0004.m0001). Protein sequences similar to *BpWOX11* were searched using NCBI’s BlastX alignment tool. The homologous genes of *BpWOX11* were identified using NCBI (https://www.ncbi.nlm.nih.gov/), and the neighbor joining phylogenetic tree was constructed using Clustalx 1.83 (Conway Institute UCD Dublin) [20] and MEGA7.0 (Molecular Evolutionary Genetics Analysis) [21].

### Construction of the overexpression vector

The full-length CDS sequence of *BpWOX11* was amplified by PCR from pCloneEZ-*BpWOX11* and ligated into p35S: *BpWOX11* overexpression vector by homologous cloning (2 × 35 S driving *BpWOX11*, gene terminator is CaMV term, resistance gene is Bar, resistance gene promoter is 35 S, resistance gene terminator is 35 S PolyA) (Catalog. No. VK011-06, Viewsolid Biotech, Beijing, China). Table S1 lists the primer sequences. T4 ligase was used based on the requirements of the vector construction kit. Constructs were incubated overnight at 16 °C. The positive recombinant plasmid (p35S:: *BpWOX11*) were screened from Glyphosate (5 mg / L) resistance plates.

### Genetic transformation of *BpWOX11*

After sequencing and confirming the correct sequence, the positive recombinant plasmid (p35S:: *BpWOX11*) was transformed into *Agrobacterium* EHA105 using the electric shock method to obtain the EHA105 (p35S::*BpWOX11*) engineered bacteria [22]. Transgenic birch plants were obtained via the *Agrobacterium*-mediated transformation of birch zygotic embryos. Open pollinated seeds were collected from clonal trees (one genotype) in seed orchard of Betula pendula in Northeast Forestry University. A bulb contains a set of seeds, each set of seeds is dried and sealed in a plastic bag and stored in a refrigerator at − 20 °C.The wild type birch (seeds from one bulb for transgenic experiment) seeds were immersed in water for 2 or 3 days, and sterilized in 30% hydrogen peroxide for 15 min and rinsed with sterile water, before seeds were prepared to be used as the transgenic explants. *Agrobacterium tumefaciens* containing the p35S:*BpWOX11* vector was used to infect the longitudinally cut seeds. Eventually, the explants were placed onto co-culture medium (WPM + 0.8 mg/L 6-BA, 0.02 mg/L NAA) under dark for 2 days and then planted on selective medium (WPM + 0.8 mg/L 6-BA, 0.02 mg/L NAA, 5 mg / L glyphosate and 200 mg/L cefotaxime). After 30 days, resistant adventitious buds were induced (WPM with 0.8 mg/L 6-BA, 0.02 mg/L NAA, 0.5 mg/L GA3,5 mg / L glyphosate and 200 mg/L cefotaxime) and rooted (WPM + 0.4 mg/L IBA), half of the uninfected seeds were cultured in initial culture medium (WPM + 0.8 mg/L 6-BA, 0.02 mg/L NAA) for 32 days and finally in induction medium (WPM + 0.8 mg/L 6-BA, 0.02 mg/L NAA and 0.5 mg/L GA3) for 32 days to obtain the WT line [22]. The composition of the medium is listed in Table S2. The temperature of the tissue culture room was maintained at 25 °C ± 2 °C, a photoperiod of 16/8 h and 1000–1500 lx. The DNA from transgenic and WT birch lines was extracted by using extraction kit (DNAquick Plant System, TIANGEN, Beijing, China). Transgenic lines were identified through PCR and primers are shown in Table S1. The primer sequence were designed using specific sequences based on p35S:*BpWOX11*, which include one end of the gene and part of the vector [22].

### Quantitative reverse transcriptase-PCR (qRT-PCR) of transgenic birch lines

Total RNA was extracted from leaf of transgenic plants and WT lines using the RNeasy Plant Mini Kit (Biotake) and reverse transcribed into cDNA using the ReverTreAce® qPCR RT Kit (Toyobo, Osaka, Japan). The SYBR Green PCR master mix (Toyobo) was used for qRT-PCR in the ABI 7500 Real-Time PCR system. Bp18S rRNA and BpActin was used as the internal reference gene, and gene-specific primers were designed using the Oligo7 software (Table S3). The results were analysed using the 2^−ΔΔCT^ method. Each reaction was repeated three times [22].

### AR growth of transgenic plants under tissue conditions

Three transgenic lines (OE3, OE6, and OE8) were selected for rooting experiments. Eighteen plants with similar growth statuses were selected from each line for rooting culture in rooting experiment. Six plants were placed into each bottle containing rooting medium. To investigate the rooting ability of the transgenic *BpWOX11* plants, the rooting medium was deprived of indole-3-butyric acid. The rooting rate was measured at 14 and 28 days. The number and length of the ARs of the three OE and WT lines were determined at 28 days.

### Greenhouse evaluation of root cuttings experiment

One-year-old plants of OE3, OE6, OE8, and WT were planted for the cuttings experiment in the greenhouse. Cuttings without apical buds, each measuring 6–8 cm in length and having one leaf at the top, were utilized. The experiment divided into three replications, with 30 cuttings used in each replication. Automatic sprinkler irrigation system was used, maintaining the culture environment at a daytime temperature of 28 °C ± 2 °C and nighttime temperature of 22 °C ± 2 °C with 70–80% humidity. At day 30, rooting rate, as well as the number and length of the ARs were determined.

### RNA-seq and differential gene expression analysis

RNA-seq samples were collected from the bases of 0- and 15-day cuttings from OE6 and WT from greenhouse. Each treatment comprised three biological replicates and each replicate contained 30 cuttings. RNA was extracted by CTAB method from 12 samples [23]. The quality of RNA was detected by 1% agarose gel, NanoDrop 2000, and Agilent 2100 Bioanalyzer. Oligo (DT)—magnetic beads were used to enrich poly (a) mRNA. Fragment buffer was used to fragment the poly (a) mRNA. The fragments were reverse transcribed into cDNA library using test kit (Illumina, San Diego, United States). Read mapping, quality control, and functional annotation were obtained from the BGISEQ-500 platform. Low-quality reads, defined as these with more than half of the component bases in the reads having a quality score of < 15, were filtered out using SOAPnuk (v1.4.0; parametric-l: 15 -q 0.2 -n 0.1) software. Other low-quality regions such as adapter sequences and regions with an excessive number of unknown bases (annotated as “Ns”) were also filtered by SOAPnuk. The remaining clean reads were mapped onto the birch reference genome (https://genomevolution.org/coge/api/v1/genomes/35080/sequence) using the Hierarchical Indexing for Spliced Alignment of Transcripts HISAT (v2.1.0) system [24]. Bowtie2 was used to align clean reads to the reference coding genomic sequence and the fragments per kilobase of transcript per million mapped reads (FPKM) was calculated by RSEM (v1.2.12) [25]. Differentially expressed genes (DEGs) were identified by DEseq2 (v1.4.5) with the criteria of | log2 Fold Change| ≥ 1, and a false discovery rate (FDR) <0.05 [26]. Gene annotation used NCBI/Nr (http://www.ncbi.nlm.nih.gov), NCBI NT (http://www.ncbi.nlm.nih.gov), Swiss-Prot (http://www.uniprot.org/keywords/), and Kyoto Encyclopedia of Genes and Genomes (KEGG) pathway database (http://www.genome.jp/kegg). The target genes were annotated in the Gene Ontology (GO) database (http://www.geneontology.org/) and then classified into their corresponding functional categories for study of biological functions.

### Data processing and statistical analysis

One-way or two-way analysis of variance (ANOVA) was performed for multiple comparisons using Student t-test or Tukey’s test. All results are expressed as the mean ± standard error of the mean with statistically significant at *p* < 0.05 (*), *p* < 0.01 (* *), *p* < 0.001 (* * *), *p* < 0.0001 (* * * *). TBtools [27], SPSS22.0, and GraphPad Prism (GraphPad Prism 8; GraphPad) were used to analyze and plot the obtained data.

## Results

### *BpWOX11* overexpression lines were obtained by genetic transformation

**Fig. 1:**
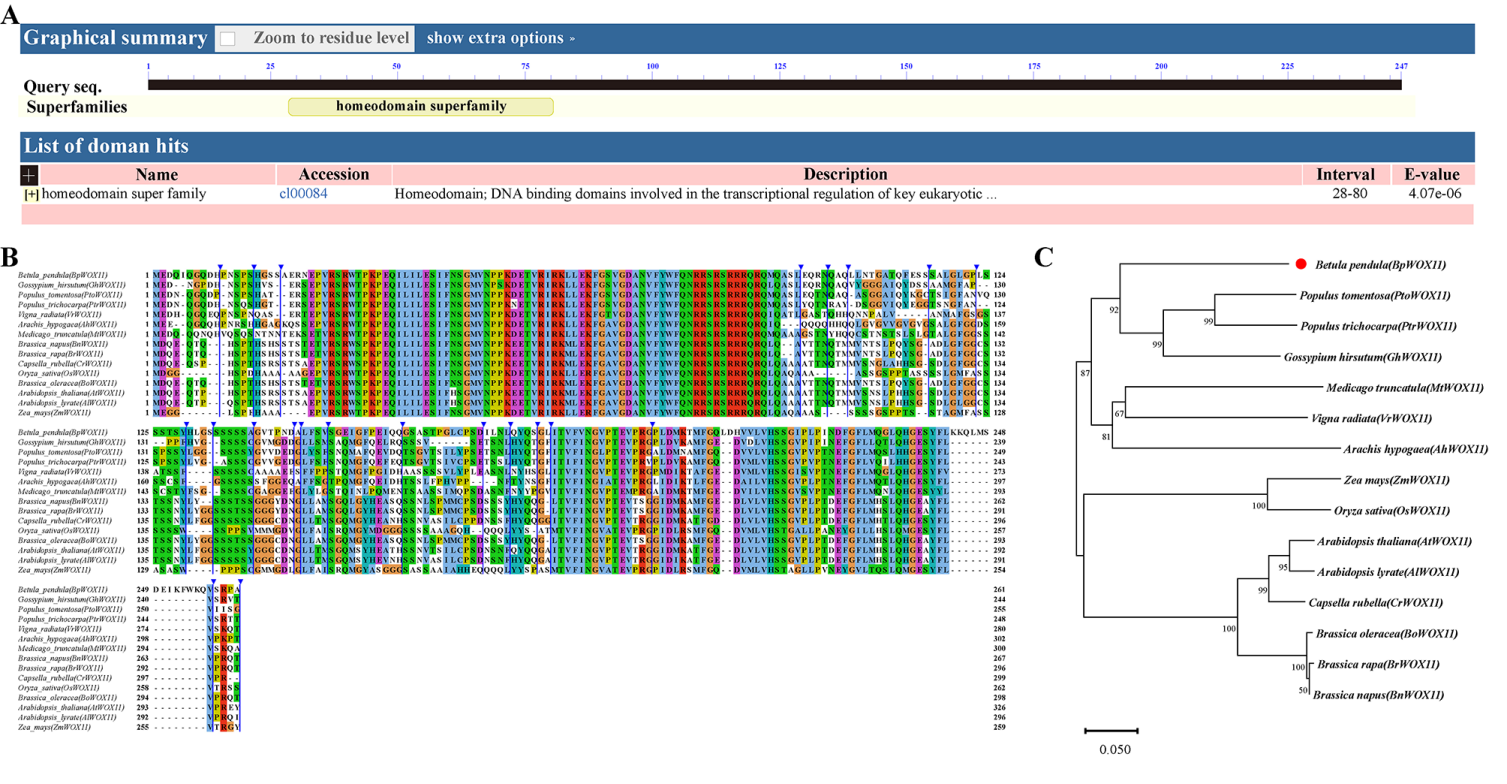
Sequence and phylogenetic analysis of *BpWOX11*. (**A**) BlastX analysis of the *BpWOX11* sequence; (**B**) *BpWOX11* amino acid sequence alignment with other that of *WOX11* other species; (**C**) *BpWOX11* and *WOX11* amino acid sequence clustering analysis of different species

In the birch genome, Bpev01.c0441.g0004.m0001 was identified as *BpWOX11*, a homologous gene of *AtWOX11* in *Arabidopsis thaliana*, with a similarity of 49.31%. The CDS of *BpWOX11* was 744 bp, encoding a protein of 247 amino acids, belonging to the homeodomain superfamily family (Fig. 1A). *BpWOX11* CDS amplification showed that the amplified sequence was of 750 bp (Fig S1). After the purification, ligation, and transformation of *E*. *coli*, a single clone was selected for sequencing. The results showed that the amplified sequence was 744 bp, consistent with the results obtained using the NCBI database. Further protein sequence alignment and phylogenetic tree analysis of *BpWOX11* were performed (Fig. 1B and C). *BpWOX11* showed high similarity to *WOX11* across several species, with the highest homology, 64.50% with *GhWOX11* of cotton.

**Fig. 2:**
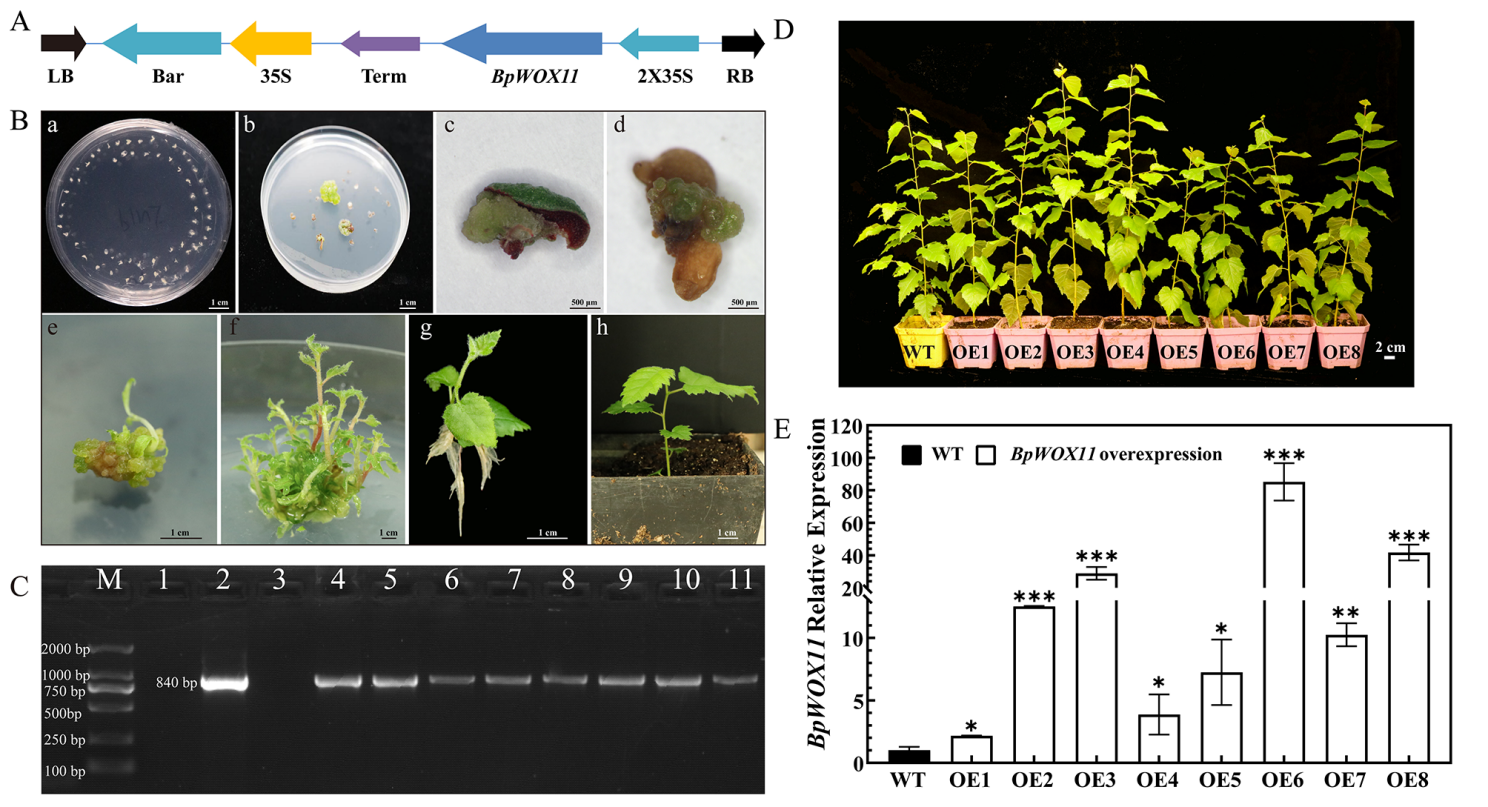
Acquisition and identification of birch DNA and RNA containing transgenic *BpWOX11*. (**A**) p35S::*BpWOX11* vector map; (**B**) genetic transformation, a: co-cultivation medium; b: selective medium; c: callus produced from the wt embryo; d: resistant callus produced from the zygotic embryo; e: adventitious buds differentiated from callus; f: transgenic cluster of shoots; g: the rooting culture of resistant clusters of shoots; h: transplanted plants; (**C**) PCR results of the overexpression (OE) lines: M: DNA marker, DL 2000; 1: negative control (water); 2: positive plasmid (p35S::*BpWOX11*); 3: Wild-type (WT); 4–11: OE1–OE8 lines(full-length gels are presented in Supplementary Fig. 2); (**D**) Transgenic lines and WT in ten-month-old; (**E**) Gene expression of *BpWOX11* among transgenic lines and WT. Mean with statistically significant was at *p* < 0.05 (*), *p* < 0.01 (* *), *p* < 0.001 (* * *)

*BpWOX11* OE lines (OE1–OE8) were obtained by *Agrobacterium*-mediated zygotic embryo transformation (Fig. 2A and B). DNA was extracted from leaves of ten-month-old transgenic *BpWOX11* overexpression lines (OE1-8) and control (WT). The positive plasmid and eight transgenic lines amplified a single band near 750 bp, which was consistent with *BpWOX11* sequence adding a section of vector sequence length (840 bp). The WT DNA did not show an amplified band (Fig. 2C). The WT and eight (OE1–OE8) positive lines are shown in Fig. 2D.

RNA was extracted from leaves of ten-month-old transgenic *BpWOX11* overexpression lines (OE1-8) and control (WT). Gene expression of *BpWOX11* in OE1–OE8 lines was significantly higher than that in WT lines (*p* < 0.05). OE6, OE3, and OE8 lines was 84.14, 40.69, and 27.86 times higher than that in WT lines, respectively (Fig. 2E). The three lines were selected for subsequent experiments.

### *BpWOX11* improves AR formation and rooting rate for propagation by cuttings

**Fig. 3:**
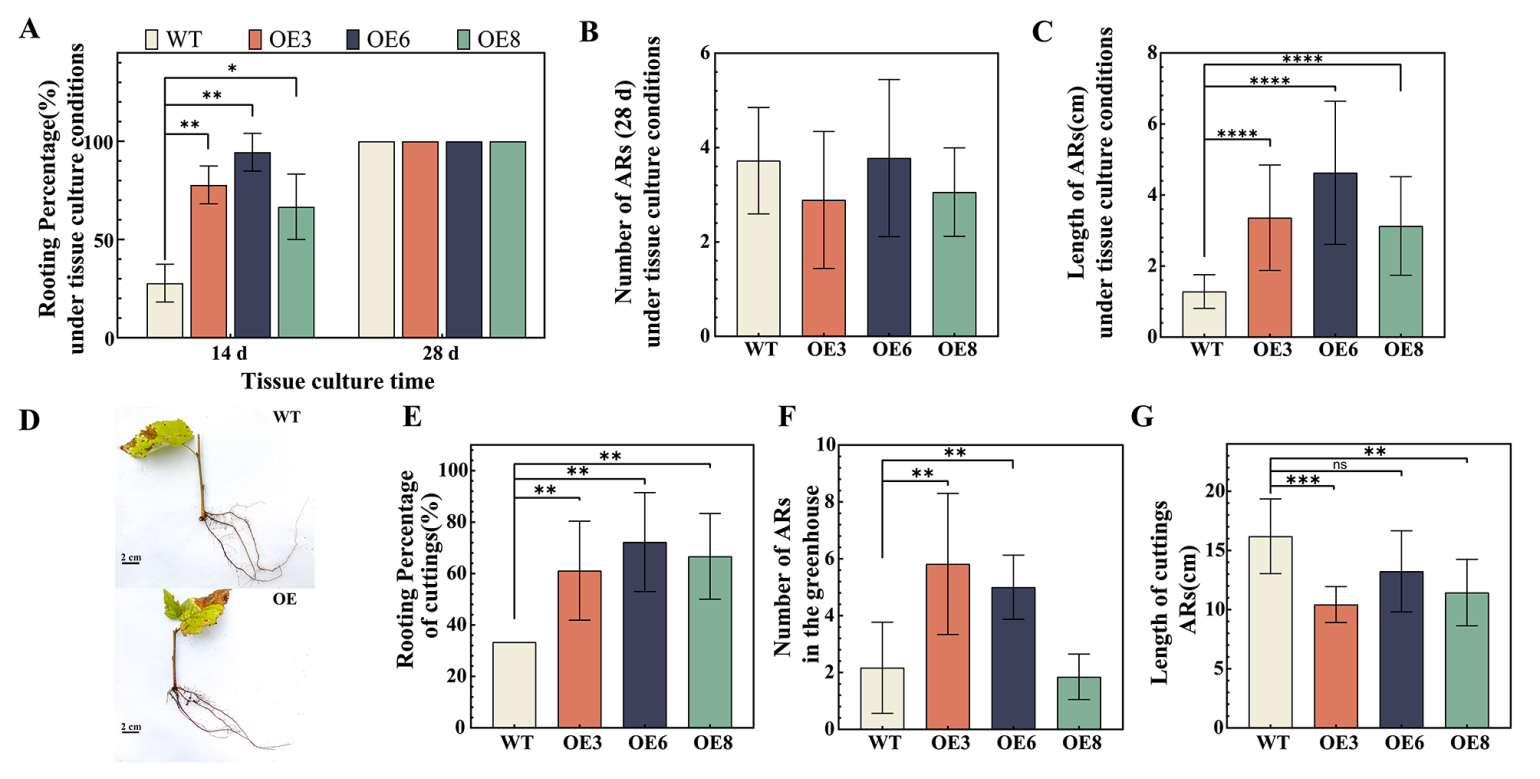
Rooting of transgenic BpWOX11 birch. (**A**) Rooting rate of cuttings at different time points under tissue culture conditions; (**B**) Number of ARs at day 28 under tissue culture conditions; (**C**) Root length of cuttings at day 28 under tissue culture conditions; (**D**) Photographs of rooted cuttings of OE and WT lines in the greenhouse; (**E**) Rooting rate of cuttings in the greenhouse; (**F**) Number of ARs in the greenhouse; (**G**) Length of adventitious roots in the greenhouse. Mean with statistically significant was at *p* < 0.05 (*), *p* < 0.01 (* *), *p* < 0.001 (* * *), *p* < 0.0001 (* * * *)

Measurements of AR growth under tissue culture conditions showed that the rooting rate of the OE lines was significantly higher than that of the WT lines without exogenous hormone application at day 14 (Fig. 3A). No significant difference was observed in the rooting rate and AR number between the OE and WT lines at day 28 (Fig. 3A and B). However, AR length of OE lines was significantly higher than that of the WT lines (Fig. 3c). At day 30, growth rates relative to pre-rooting (0d) were significantly higher for the OE2, OE5, and OE7 lines than for the WT line, whereas no significant differences were observed for the other transgenic lines (Fig S3).

In the greenhouse cuttings experiment (Fig. 3D), results show the rooting rate was 33.33% for WT, whereas OE3, OE6, and OE8 lines were 61%, 72% and 67%, respectively (Fig. 3E). The number and length of AR for OE6 and OE3 lines were significantly higher than that in the WT lines. The root lengths for OE3 and OE8 lines were significantly lower than that of the WT lines (Fig. 3F and G).

### RNA-seq analysis

**Fig. 4:**
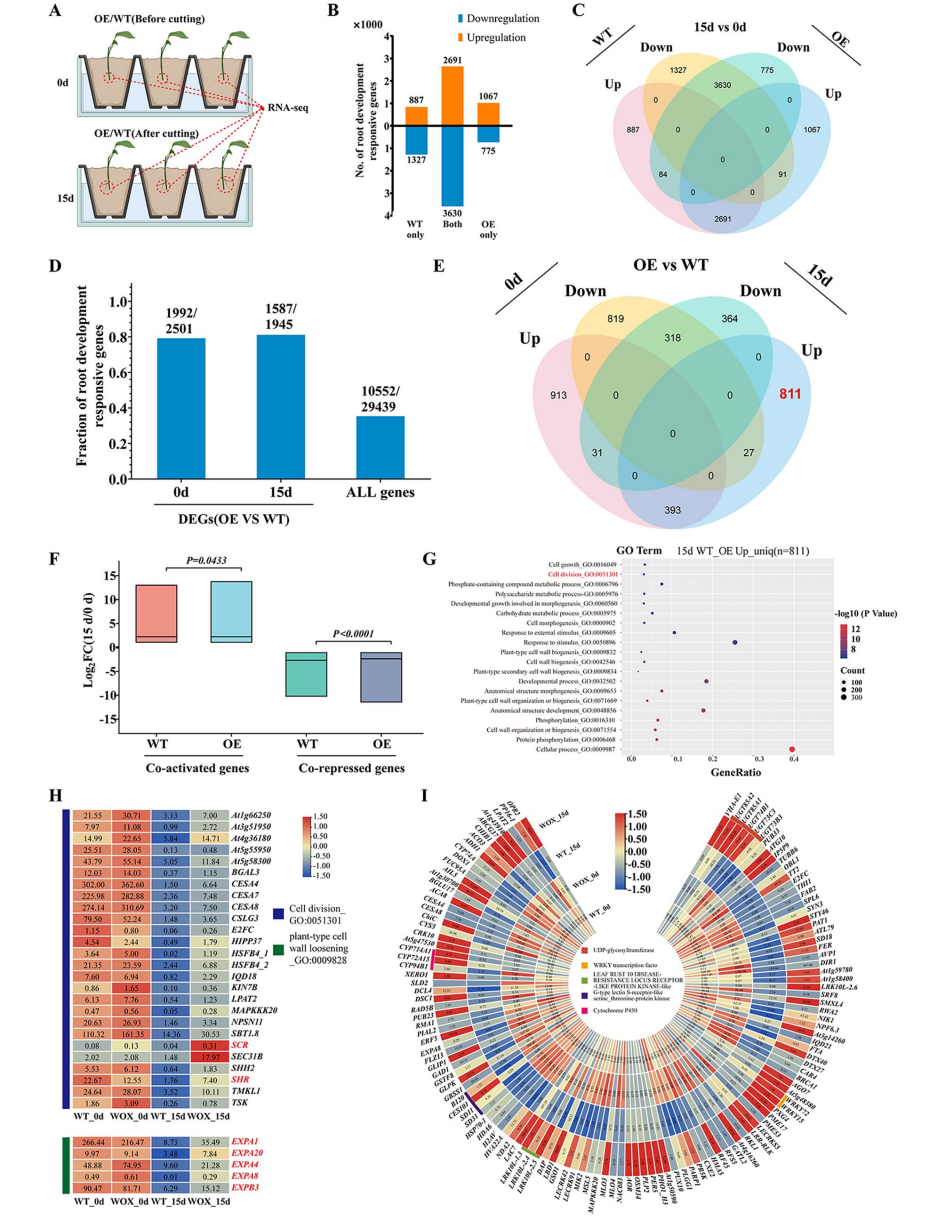
Transcriptome sequencing of overexpression (OE) and wild-type (WT) cuttings. (**A**) Schematic diagram of the cuttings (Created By BioRender.com); (**B**) The number of upregulated and downregulated of differentially expressed genes (DEGs) for the OE and WT lines in the cuttings process; (**C**) Overlapping DEGs between WT and OE lines in the cuttings process; (**D**) DEGs related to rooting-related genes for OE and WT lines. The Y-axis shows the percentage of rooting related genes before and after cuttings; (**E**) Upregulated or downregulated rooting-related genes before and after cuttings for OE lines; (**F**) The log_2_ expression ratio of the co-activation or co-inhibition genes for OE and WT lines at day 15/day 0; (**G**) Interval-based GO enrichment analysis; (**H**) Heatmap of DEGs in cell division and plant cell wall loosening pathways; I Heatmap of DEGs in response to stress pathways

The expression *BpWOX11* gene increased leading to elevated rooting rate of OE lines in birch cuttings. The bases of cuttings from OE6 and WT lines with the highest rooting rate were used. RNA-seq analysis was performed at two timepoints, day 0 and day 15, and three biological replicates were established (Fig. 4A). We identified 8770 and 8338 differentially expressed genes (DEGs) before and after cuttings in the WT and OE lines, respectively. These DEGs were called AR formation genes. Among them, 3662 were upregulated and 5048 were downregulated in the WT lines, whereas 3849 were upregulated and 4489 were downregulated in the OE lines. A large proportion of the upregulated (57.93%) and downregulated (63.33%) genes was shared between the WT and OE lines (Fig. 4B and C). The proportion of AR-related DEGs shared between the OE and WT lines increased by 79.65% and 81.59%, respectively, before cuttings (day 0) and during cuttings (day 15) (Fig. 4D). In the OE lines, up-regulated (393/1337) and down-regulated (318/1159) did not change significantly at day 15 (Fig. 4E). Results indicated that gene expression induced AR formation was conserved in the OE and WT lines.

Cuttings also induced a specific set of up-regulated (811, 65.88%) and down-regulated (364, 51.05%) genes between WT and OE lines (Fig. 4E). At day 15 vs. day 0, upregulated genes were 1067 and 887 for OE lines and WT lines, whereas downregulated genes were 775 and 1327 for OE lines and WT lines, respectively (Fig. 4B). The average gene expression for OE lines was higher than that in the WT lines (*p* < 0.05) (Fig. 4F, Table S4). These data indicated that high *WOX11* expression strongly responded to changes in gene expression during AR formation. The GO enrichment analysis showed that 811 upregulated genes and 364 downregulated genes were identified for the OE lines after rooting (Fig. 4G, Table S5 and S6). The upregulated genes were mainly enriched in cell division, cell wall biosynthesis, developmental processes, and cell morphology. The cell division pathway included genes that promoted AR formation and development, namely SCRSCARECROW (*SCR*) and SHORT ROOT (*SHR*) (Fig. 4H). *WOX11* upregulated the expression of a group of expansin genes in the plant cell wall loosening pathway (Fig. 4H). In addition, GO enrichment showed that a specific set of up-regulated genes (811 genes) between OE and WT were also enriched in the response to stress pathway after rooting. The genes enriched in this pathway mainly consisted of five gene families related to stress response, i.e., UDP-glycosyltransferase, WRKY transcription factor, LEAF RUST 10 DISEASE-RESISTANCE LOCUS RECEPTOR-LIKE PROTEIN KINASE, G-type lectin S-receptor-like serine_threonine-protein kinase, Cytochrome P450(Fig. 4I). qRT-PCR showed that rooting-related transcription factors (SCR and SHR) in the cell division pathway were consistent with the RNA-seq results (Fig S4). The activation of cell division-related genes and expansins after *WOX11* OE may increase the occurrence of ARs during birch cuttings, thereby improving the rooting efficiency of the cuttings.

## Discussion

### *BpWOX11* plays a role in the early rooting stage

Rooting rate, root length, and root number have been used as indicators to evaluate vegetative propagation [28–31]. In this study, *WOX11* overexpression led to improve the rooting rate of birch cuttings, as results of the early initiation of ARs and increase number of Ars. In *Arabidopsis*, *WOX11* OE has been reported to significantly increase the number of adventitious roots and decrease the length of main roots [9, 14]. *WOX11/12* plays a major role in the first event of rooting cell establishment. The expression of *WOX11/12* was decreased in the second step of cell fate transition, whereas those of *WOX5* and *WOX5/7* increased [32]. Therefore, we speculate that *BpWOX11* plays a major role in the early stage of *de novo* organogenesis in birch. Our results showed that overexpression *BpWOX11* accelerated the formation of new ARs in cuttings and increased the number of ARs; however, it did not promote the continuous growth process after AR formation.

### *BpWOX11* promotes rooting as result of regulating genes related to cell wall tissue and cell division

Expansin, as a cell wall relaxation protein, plays a crucial role in the root development of soybean (*Glycine max*) and *Arabidopsis* [33, 34]. In *Arabidopsis*, expansin genes (*AtEXP7* and *AtEXP18*) are closely associated with the initiation of root hairs. In plants, root hairs originate from epidermal cells which are accompanied by local cell wall loosening [35]. In this study, *BpWOX11* increased rooting rate of birch. RNA-seq analysis indicated that *WOX11* induced a group of expansins in the plant-type cell wall loosening pathway. Therefore, *BpWOX11* may promote the growth of ARs by inducing the upregulation of expansin to promote cell wall loosening.

*WOX11* promoted the expression of genes associated with cell division, such as *KIN7B*, *TSK*, *SHR*, and *SCR* [36–39]. *SHR* and *SCR* are crucial for regulating the process of asymmetric cell division in root tissue [40]. SCARECROW-LIKE gene increases its mRNA levels during the first 24 h induction of root from stem cuttings in *Pinus radiata* D. Don and *Castanea sativa* Mill [41]. The expression pattern of *PrSHR* in *Pinus radiata* is similar to that of the SCARECROW-LIKE gene, and it promoted the formation and maintenance of root meristem and the cambium area of hypocotyl cuttings [42]. These results indicated that *SHR* and *SCR* play a crucial role during the earliest stage of AR formation. Therefore, we considered that *WOX11* promotes AR formation in the early stage of birch cuttings by promoting the expression of *SHR* and *SCR*.

### Overexpression of *BpWOX11* promotes the expression of stress-related genes

In *Arabidopsis*, 18% of the DEGs in the *AtWOX11* mutant plants were involved in stress response compared with those in the WT plants. [43]. In this study, transcriptome analysis of OE6 and WT strains revealed that overexpression of WOX11 directly upregulated the expression of stress-related genes. *WOX11* can control root hair development and also play a role in increasing the drought resistance of rice [16]. Therefore, *WOX11* may also be involved in regulating the expression of stress-related genes in birch. We found that 393 genes were upregulated, and 318 genes were downregulated in *BpWOX11* OE lines at day 0 and 15 (Fig. 4E). The upregulated 393 DEGs were enriched in defense response, response to stress, and stimulation (Table S7). The downregulated 318 differential genes were enriched in phosphorylation, cell recognition, phosphorus metabolism, and defense response(Table S8). Moreover, at day 15, *WOX11* alone upregulated 207 genes involved in response to stimulus and 129 genes involved in response to stress in OE lines (Table S5). Our results showed that OE lines could be used for improving the drought resistance in the early stage of cuttings without roots in birch.

### Electronic supplementary material

Below is the link to the electronic supplementary material.

**Supplementary Material 1 (Figure): Fig S1: ***BpWOX11* Amplified Electrophoresis Map. **Fig S2:*** BpWOX11* Amplified Electrophoresis Map. **Fig S3: **Determination of relative growth after 30 days rooting. **Fig S4:** qRT-PCR validation of the transcriptome data

**Supplementary Material 2 (Table): Table S1:** Construction of over-expression vector and DNA detection primer sequence. **Table S2: **Formula of birch tissue culture medium. **Table S3:** Gene ID and primers used for qRT-PCR. **Table S4: **The average gene expression for day 15 vs. day 0 Common regulation (WT/OE). **Table S5:** GO enrichment analysis of 15d WT_WOX uniq up (n = 811). **Table S6:** GO enrichment analysis of 15d WT_WOX uniq down (n = 364). **Table S7: **GO enrichment analysis of 0d WT_WOX up and 15dWT_WOX up common (n = 393). **Table S8:** GO enrichment analysis of 0d WT_WOX down and 15dWT_WOX down common (n = 318)

## Data Availability

All RNA-seq data have been archived in the NCBI Sequence Read Archive (SRA), accession number PRJNA972472.
