# Supplementary material for: BpWOX11 promotes adventitious root formation in Betula pendula
Source: BMC Plant Biol. 2024 Jan 2;24:17. doi: 10.1186/s12870-023-04703-z (PMC10759540; doi:10.1186/s12870-023-04703-z)
Supplement: Supplementary file 1 — Supplementary Material 1 (Figure): Fig S1: BpWOX11 Amplified Electrophoresis Map. Fig S2: BpWOX11 Amplified Electrophoresis Map. Fig S3: Determination of relative growth after 30 days rooting. Fig S4: qRT-PCR validation of the transcriptome data [file 12870_2023_4703_MOESM1_ESM.docx]

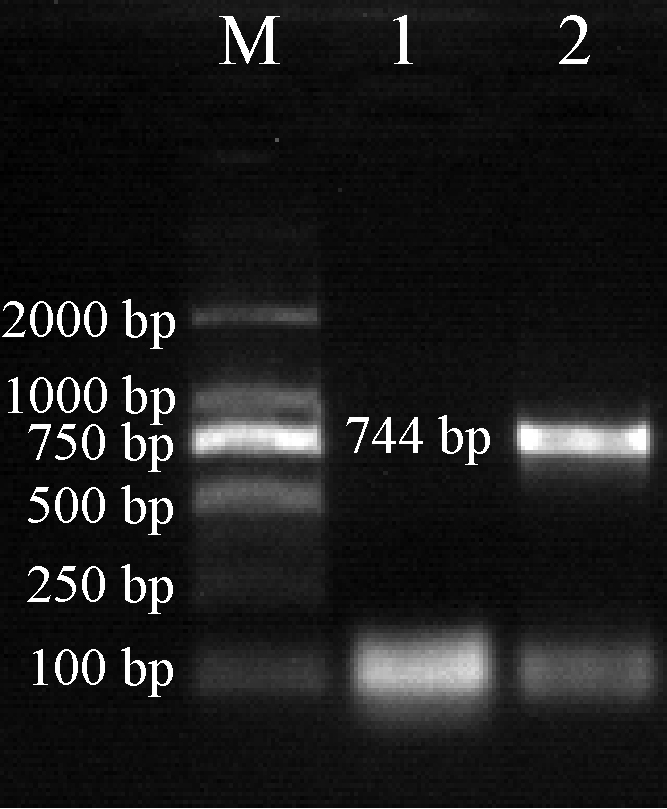

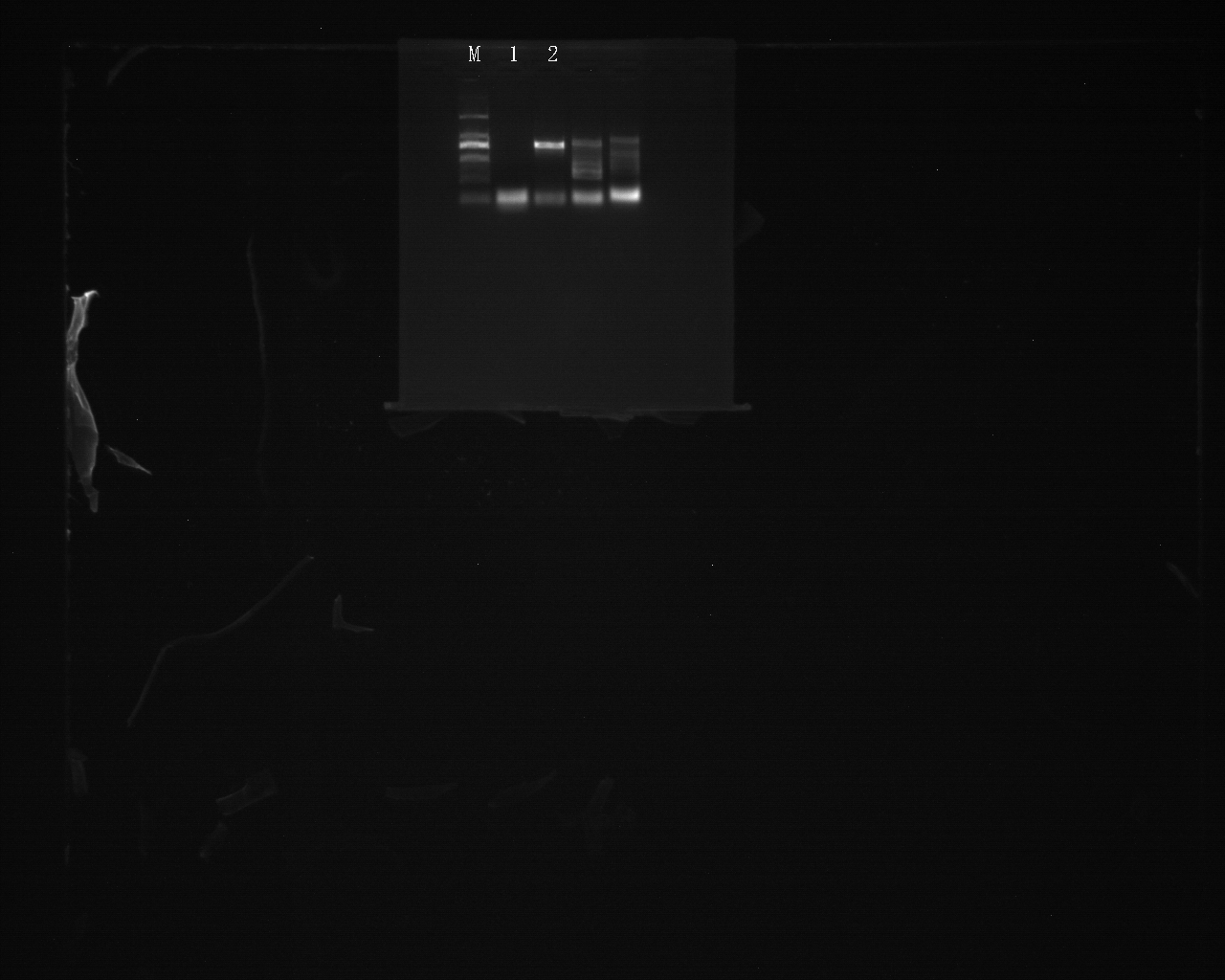


**Supplementary Figure 1.** *BpWOX11* Amplified Electrophoresis Map(left: cropped gels;right: the original, unprocessed versions.)

M: DNA Marker, DL 2000; swimming lane 1: Full length of *BpWOX11* gene.


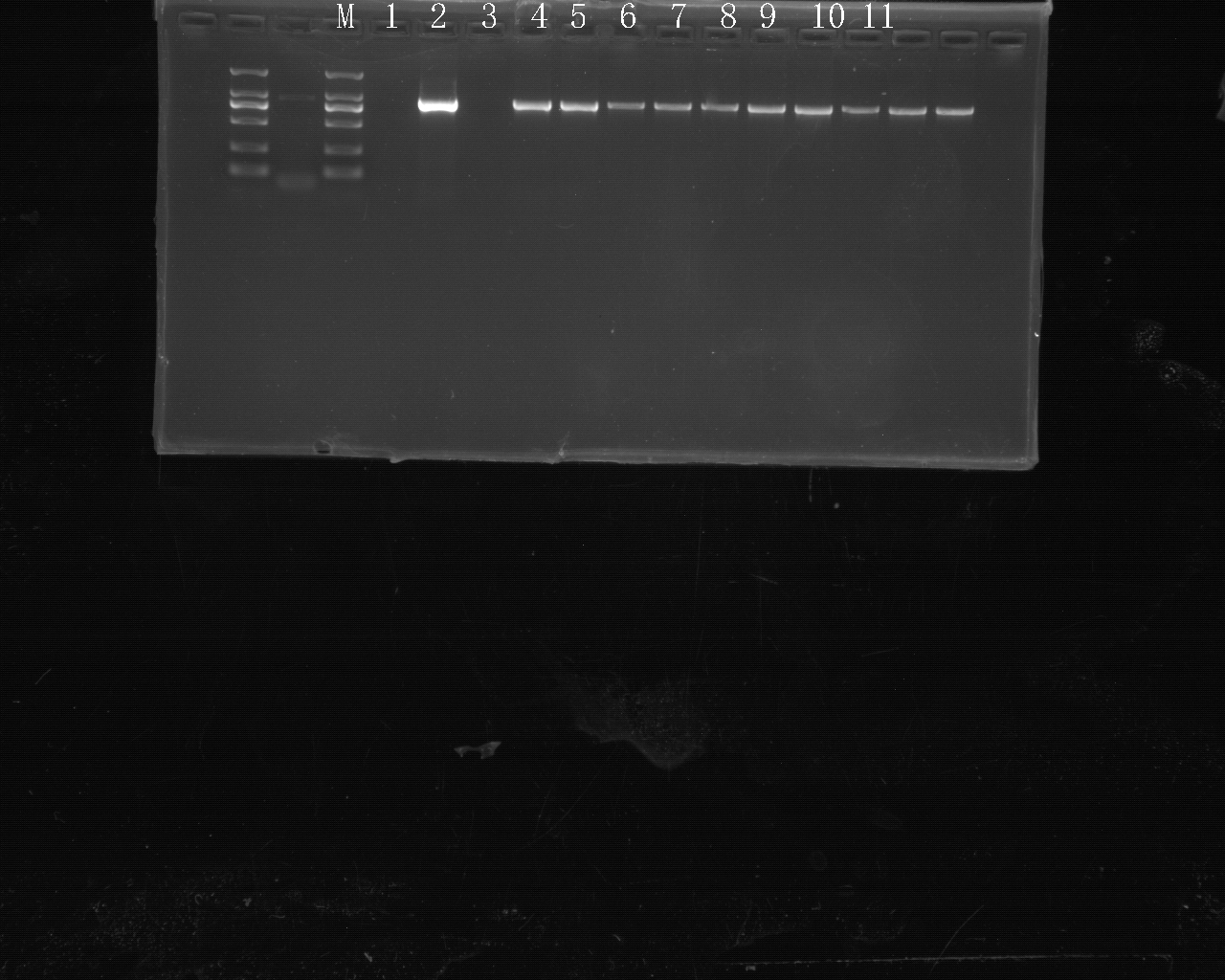


**Supplementary Figure 2.** *BpWOX11* Amplified Electrophoresis Map

PCR results of the overexpression (OE) lines of the original, unprocessed versions: M: DNA marker, DL 2000; 1: positive plasmid; 2: negative control (water); 3: Wild-type (WT); 4–12: OE1–OE8 lines


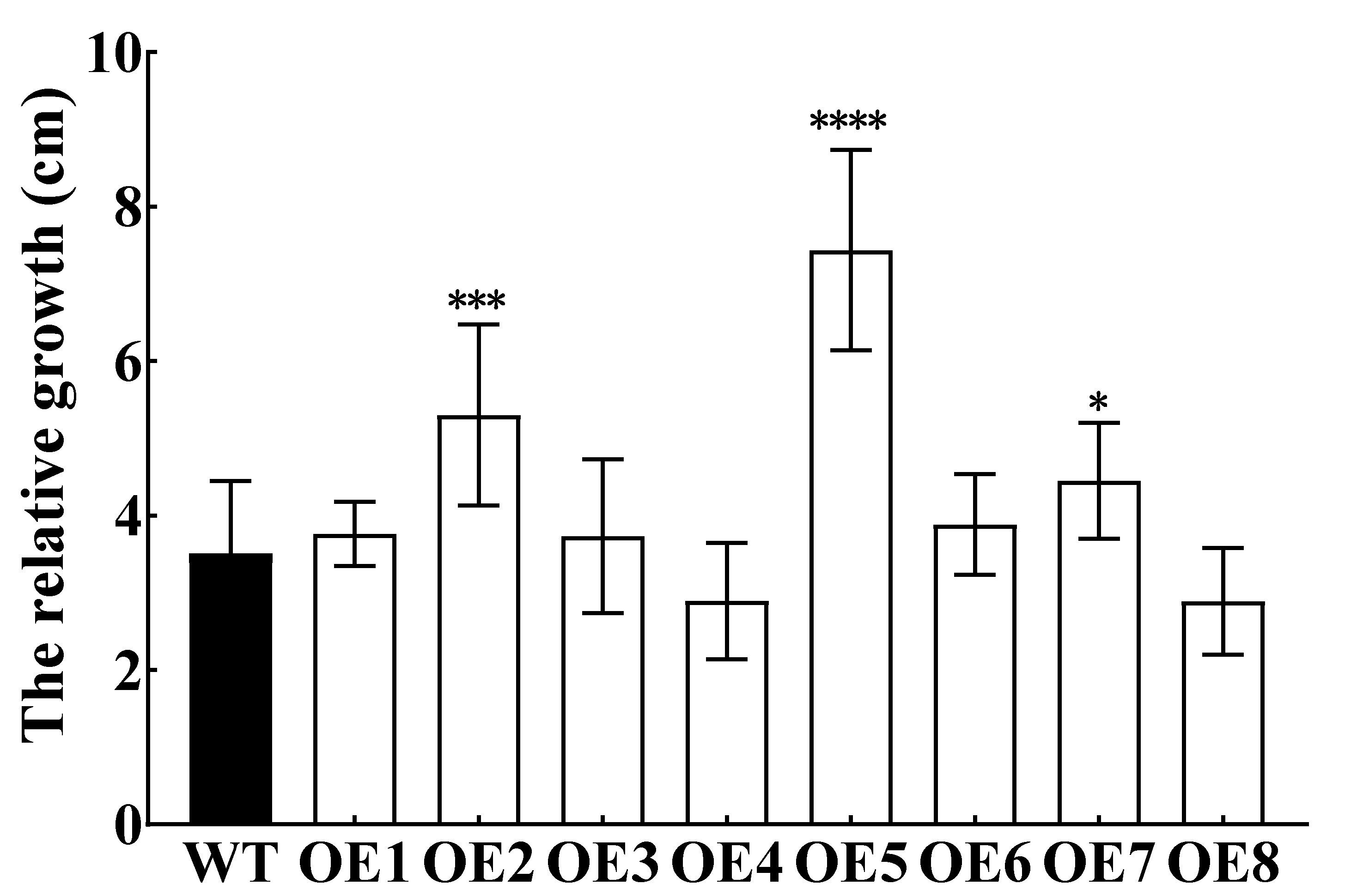


**Supplementary Figure 3.** Determination of relative growth after 30 days rooting

Mean with statistically significant was at p < 0.05 (*),p < 0.001 (* * *), p < 0.0001 (* * * *).


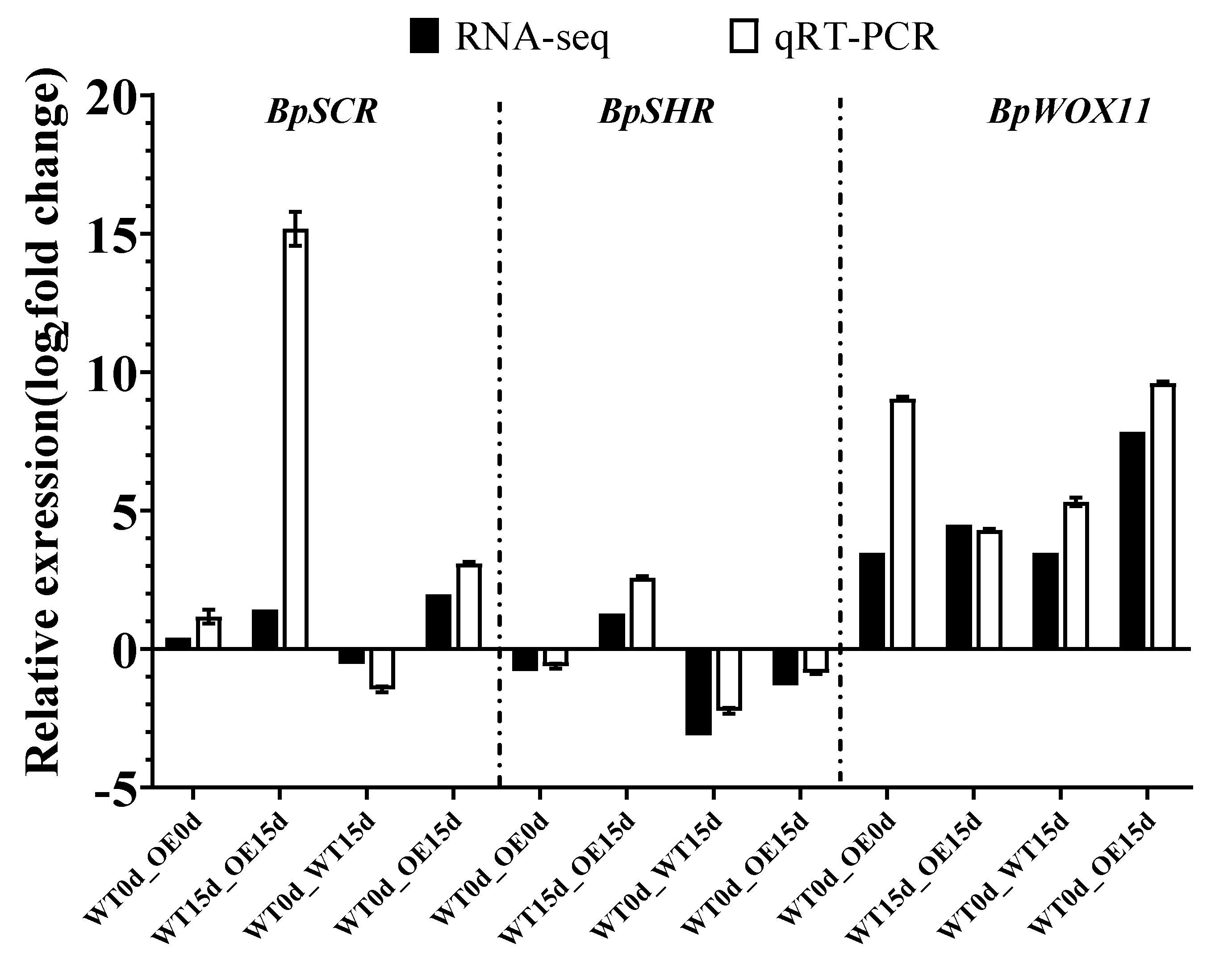


**Supplementary Figure 4.** qRT-PCR validation of the transcriptome data

Student’s t-test. Results are shown as mean expression ± standard deviation (SD) of three independent experiments.
